# Landmark Recognition in Alzheimer’s Dementia: Spared Implicit Memory for Objects Relevant for Navigation

**DOI:** 10.1371/journal.pone.0018611

**Published:** 2011-04-04

**Authors:** Roy P. C. Kessels, Amy van Doormaal, Gabriele Janzen

**Affiliations:** 1 Radboud University Nijmegen, Donders Institute for Brain, Cognition and Behaviour, Nijmegen, The Netherlands; 2 Departments of Medical Psychology and Geriatric Medicine, Radboud University Nijmegen, Medical Centre, Nijmegen, The Netherlands; 3 Radboud University Nijmegen, Behavioural Science Institute, Nijmegen, The Netherlands; University of Minnesota, United States of America

## Abstract

**Background:**

In spatial navigation, landmark recognition is crucial. Specifically, memory for objects placed at decision points on a route is relevant. Previous fMRI research in healthy adults showed higher medial-temporal lobe (MTL) activation for objects placed at decision points compared to non-decision points, even at an implicit level. Since there is evidence that implicit learning is intact in amnesic patients, the current study examined memory for objects relevant for navigation in patients with Alzheimer’s dementia (AD).

**Methodology/Principal Findings:**

21 AD patients participated with MTL atrophy assessed on MRI (mean MMSE = 21.2, SD = 4.0), as well as 20 age- and education-matched non-demented controls. All participants watched a 5-min video showing a route through a virtual museum with 20 objects placed at intersections (decision points) and 20 at simple turns (non-decision points). The instruction was to pay attention to the toys (half of the objects) for which they were supposedly tested later. Subsequently, a recognition test followed with the 40 previously presented objects among 40 distracter items (both toys and non-toys). Results showed a better performance for the non-toy objects placed at decision points than non-decision points, both for AD patients and controls.

**Conclusion/Significance:**

Our findings indicate that AD patients with MTL damage have implicit memory for object information relevant for navigation. No decision point effect was found for the attended items. Possibly, focusing attention on the items occurred at the cost of the context information in AD, whereas the controls performed at an optimal level due to intact memory function.

## Introduction

For successful navigation in our environment, information about landmarks, spatial locations and routes has to be efficiently processed. In order to do so, our neurocognitive system relies on both the dorsal ‘where/how’ system that is implicated in action-related behavior and the central ‘what’ route, necessary to process object information [1]. Another crucial brain structure involved in spatial behavior is the hippocampus. It has been suggested that the hippocampus stores spatial information in the form of an allocentric cognitive map, both in animals [2] and man [3]. Finally, there is increasing evidence that the hippocampus may act as a ‘binding device’, integrating object and spatial information from both the dorsal and the ventral streams [4].

Interestingly, navigational expertise and landmark memory are related to larger hippocampal volumes [5]. In addition, functional neuroimaging studies in healthy volunteers have demonstrated increased parahippocampal activation related to memory for objects relevant for navigation, i.e. landmarks at decision points, compared to objects that are irrelevant for navigation (objects placed at non-decision points). This latter finding was also reported for landmark objects that were not correctly recognized in a later object recognition task, indicating that object information that is relevant for navigation may be processed in an automatic way [6]–[8]. Furthermore, good navigators demonstrated higher hippocampal activation than normal navigators [9]. Conversely, lesion studies have consistently demonstrated impairments on a wide range of tasks that are relevant for spatial navigation. That is, deficits in positional memory and object-location binding have been reported in patients with unilateral hippocampal lesions [10], [11]. Also, impairments in virtual maze learning have been found in hippocampally lesioned patients [12].

While there is abundant evidence for spatial learning and memory decrements in patients with unilateral hippocampal lesions, remarkably little research has been done on spatial memory and learning in patients with Alzheimer’s dementia (AD), in which relatively selective bilateral hippocampal atrophy is consistently reported in the early stages of the disease [13]. Only a few studies have examined static object-location memory tasks in AD patients, demonstrating impaired performance compared to controls [14], [15]. Using a real-world wayfinding test, Monacelli and colleagues [16] investigated a group of AD patients and demonstrated impaired spatial navigation and spatial orientation in the AD group, possibly due to an underlying deficit in linking landmark information to route knowledge. Similar findings were also reported using virtual maze-learning paradigms in AD patients [17], [18].

With respect to landmark recognition, however, conflicting results have been found in AD. For example, some authors reported worse landmark memory in AD patients compared to matched controls [16], [19], whereas others did not demonstrate performance differences on landmark recognition between AD patients and controls [20]. In the present study, memory for objects relevant for navigation is investigated in a group of AD patients and matched-controls. Participants were presented with a film of a route through a virtual museum, containing objects placed at either decision points (i.e., a landmark) or non-decision points (no landmark). We expect that patients overall perform worse than controls, but that memory for landmark is better than memory for objects placed at non-decision points. In addition, since previous findings suggest that poor performance on spatial memory tasks was affected by attention deficits in AD patients [21], we expect that attention mediates the expected landmark effect (i.e., objects to which the participants are instructed to pay attention to will be remembered better).

## Materials and Methods

### Participants

Twenty-one AD patients (6 males, mean age 77.4, S.D.  = 6.5) were recruited from the memory clinic of the Lievensberg Hospital in Bergen op Zoom, the Netherlands. All patients fulfilled the criteria for probable or possible AD according to the NINCDS-ADRDA criteria [22]. The clinical diagnosis was made applying a multidisciplinary approach using a clinical interview, medical examination, or neuropsychological assessment. Also, medial-temporal lobe atrophy (MTA) was visually rated by a neuroradiologist using 1.5T structural coronal magnetic resonance images (MRI) using the Scheltens rating scale [13], which has an established validity in comparison with volumetric measures [23]. MTA ratings indicated on average a moderate degree of MTA (mode 2, range 0–4). Mean performance on the Mini-Mental State Examination (MMSE) [24] for the patients was 21.1 (S.D.  = 4.0). Twenty age-matched healthy controls (6 males; mean age  = 75.0, S.D.  = 5.8) were recruited from the same geographic region as the hospital. None of the healthy participants had a history of neurological or psychiatric disease or used psychopharmacological drugs, reported subjective memory impairment or showed cognitive decline on the MMSE (mean 27.3, S.D.  = 1.6). Exclusion criteria for all participants were visual or motor impairments that could not be compensated for and communication problems (i.e., aphasia, visual agnosia or inability to communicate in Dutch). All included participants completed the study and were included in the eventual analyses. Premorbid intelligence was assessed using the Dutch version of the National Adult Reading Test (NART) [25]; mean estimated IQ for the AD group was 88.1 (S.D.  = 13.5) mean estimated IQ for the controls 87.2 (S.D.  = 11.4). No group differences were found with respect to age (*t*_(39)_  = 1.3), premorbid intelligence (*t*_(38)_  = 0.2) and sex distribution (Mann-Whitney *U* = 207.0, *Z* = 0.1).

### Ethics Statement

Medical-ethical approval was obtained from the Institutional Review Board of the Lievensberg Hospital in Bergen op Zoom and written informed consent was obtained in all participants, in accordance with the declaration of Helsinki.

### Apparatus

**Figure 1:**
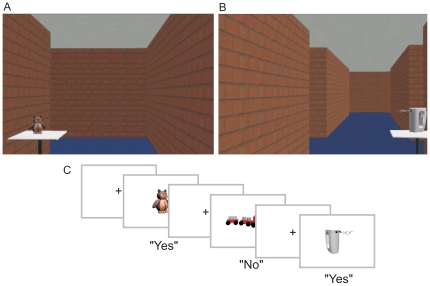
Stills from the virtual museum route showing a toy placed at a non-decision point (A) and a non-toy at a decision point (B), and schematic overview of the recognition trials (C). Participants had to indicate whether the presented object was shown in the movie or not by button press.

A computerized paradigm was used to study memory for navigationally relevant objects [6]. Commercially available software (3D TraumhausDesigner 4.0, Data Becker GmbH & Co.KG) was used to construct a film sequence through a virtual-reality museum (see Figure 1). The virtual museum consisted of a maze with 40 three dimensional colored objects presented on tables along the route. Half of the objects were presented at decision points (a place on the route with more than one directional option, e.g., a left or right turn), half were presented at non-decision points (i.e., along the route at a location with only one directional option, e.g. a left turn). It should be noted that the participants themselves did not have to make a choice about the direction, but the term decision point is used more broadly to indicate a point in an environment with multiple directional possibilities [6], [26]. Half of the objects were toys, and the other half were objects from other semantic categories. Participants were explicitly instructed to pay special attention to the toys. All four sets of objects (decision-point objects toys, decision-point objects non-toys, non-decision point objects toys, non-decision point objects non-toys) were matched for word frequency using the CELEX database of the Max Planck Institute for Psycholinguistics (http://celex.mpi.nl/). The range from low to high frequent objects (i.e. reflecting “difficult” and “easy” items as a measure of familiarity) was equal in all object sets, and there were no statistically significant differences in word frequency between all four sets of objects (all *t*-values ≤1.0). Length of the film was 5 minutes and 14 seconds and the same film was used for all participants. The film was presented at a fixed speed on a 1.60 GHz-M Pentium 4 laptop with 512 Mb RAM with a 15.0 inch LCD screen. A button box with a “YES” and a “NO” button was used to measure the responses.

### Procedure

All participants were instructed that they were going to see a film of a virtual maze with objects placed on tables, representing a virtual museum. They had to read the names of all objects aloud and try to remember the objects. In case participants could not name a specific object, its name was given by the experimenter. In addition, they had to pay special attention to objects that were interesting to children (i.e., the toys). Toys were equally placed at decision and non-decision points to control for attention effects. Furthermore, they were told to pay attention to the direction of the route. Also, the participants were told that the film would be presented twice. After the second presentation of the movie, a recognition test followed in which objects were presented in the middle of the computer screen (one at a time) and the participants had to indicate whether this object was presented in the maze or not by button press. First, a practice trial was shown to make the participants familiar with the procedure (using 4 objects that had not been presented in the maze). Next, the actual test followed with 80 objects, 40 of which that had been presented in the maze and 40 new objects that had not been presented, but had the same appearance as the presented objects. All objects were presented in a different random order for each participant.

### Analyses

Both accuracy and response times were measured. For each group separately, trials with outlier response times were removed from further analyses. A restricted-mean analysis was performed and response times more than 2 standard deviations above the trial mean of the respective group were considered outliers, that is, invalid responses. Applying Chauvenet’s criterion of 2 standard deviations indicates a probability of 2.3% or less that the outlier response actually originates from the same statistical distribution as the other observations [27]. For the controls, the outlier rate was 5.7%, for the patients this was 6.4%. Response times were analyzed only for the correct responses. With respect to the accuracy data, the overall nonparametric discrimination index A′ [28] was computed, taking overall hits and false alarms into account (all stimulus types taken together compared with distracter items). Subsequently, accuracy and response times were analyzed separately using a repeated-measures General Linear Model (GLM) analysis of variance with Attention (toys vs. non-toys) and Landmark (decision-point vs. non-decision point) as within-subject factors and Group (controls vs. AD) as between-subject factor. Accuracy and response times for the distracter items were compared using *t*-tests. One-sample *t*-tests were performed for the controls and patients separately to determine whether the accuracy for each stimulus type was significantly higher than chance performance (0.5). Correlations (Spearman's ρ) were computed between the MTA ratings and the recognition performance in the patients (α set at 0.01).

## Results

**Figure 2:**
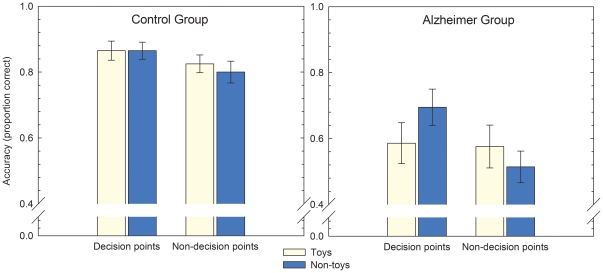
Recognition accuracy (+S.E.M.) for the controls and the Alzheimer group for the toys and non-toys placed at decision points or non-decision points.

Figure 2 shows the accuracy data for the controls and AD patients for the toy and non-toy objects placed at decision points or non-decision points. Overall, the controls had a higher discrimination index (*A*′ = 0.93, *S.E.M.*  = 0.01) than the Alzheimer patients (*A*′ = 0.74, *S.E.M.*  = 0.03), reflecting an overall worse performance in the Alzheimer group (*t*_(39)_  = 5.9, *p*<0.0005). Repeated measure GLM analysis on the accuracy data showed a main effect for Group (*F*_(1,39)_  = 19.3, *p*<0.0005) and Landmark (*F*_(1,39)_  = 13.6, *p*<0.001). No main effect for Attention was found (*F*_(1,39)_  = 0.06). In addition, a significant interaction between Attention and Landmark was found (*F*_(1,39)_  = 8.4, *p*<0.006), as well as a significant interaction for Attention × Landmark × Group (*F*_(1,39)_  = 4.7, *p*<0.04). No significant interactions were found for Landmark × Group (*F*_(1,39)_  = 1.1) or Attention × Group (*F*_(1,39)_  = 0.6). Post-hoc *t*-tests (one-tailed) demonstrated higher accuracy for non-toys placed at decision points compared to non-decision points, both for the controls (*t*_(19)_  = 1.7, *p*<0.05) and the Alzheimer patients (*t*_(20)_  = 4.5, *p*<0.0005). No landmark effect was found for the toy objects (controls: *t*_(19)_  = 1.0, patients: *t*_(20)_  = 0.3). Furthermore, non-toys placed at decision points significantly differ from toys at decision points for AD patients but not for the controls (*t*_(19)_  = 2.5, *p*<0.05). With respect to the distracter items, recognition accuracy for the controls (*M* = 0.91, *S.E.M*.  = 0.01) and the Alzheimer patients (*M* = 0.71, *S.E.M.*  = 0.06) differed significantly, with the patients performing worse than the controls (*t*_(39)_  = 3.4, *p*<0.002). Recognition performance for all stimulus types was above chance for the controls (all t_(19)_-values >3.2, *p*<0.001). For the AD patients, recognition accuracy was significantly above chance for the non-toys placed at decision points (t_(20)_  = 3.6, *p*<0.001) and for the distracter objects (*t*_(20)_  = 28.0, *p*<0.0005). A trend towards an above-chance performance was found for the toys placed at decision points (*t*_(20)_  = 1.4, *p* = 0.09). Performance did not differ statistically from chance level for the objects placed at non-decision points (toys: *t*_(20)_  = 1.2; non-toys: *t*_(20)_  = 0.3).

Table 1 shows the response time date for the controls and Alzheimer patients for the toy and non-toy objects placed at decision points or non-decision points. A significant main effect for group was found (*F*_(1,37)_  = 12.8, *p*<0.001), with the Alzheimer patients performing slower on the task than controls, in agreement with what is typically found [29]. No other main effect or interaction effects were statistically significant (all *F*-values <3.1). With respect to the correlation between task performance and MTA rating in the patients, no significant correlations were reported (all absolute ρ-values <0.46).

**Table 1:** Mean response times (+S.E.M.) for the toys and non-toys placed at decision points or non-decision points, as well as the distracter items (correct trials only).

		Control Group		Alzheimer Group	
		*Mean*	*S.E.M.*	*Mean*	*S.E.M.*
Toys	Decision points	984.3	41.2	2405.3	397.4
	Non-decision points	1017.3	50.5	2000.4	254.6
Non-toys	Decision points	1044.6	54.3	2213.8	317.5
	Non-decision points	1100.2	81.3	2641.0	470.5
Distracter objects		1150.9	51.8	2227.8	252.5

## Discussion

In this study, we examined memory for objects relevant for navigation (i.e., landmarks at decision points) compared to memory for objects that were presented along the route, but at locations irrelevant for navigation. In agreement with previous studies in healthy young adults [30], we demonstrated better performance on recognition of landmark objects versus recognition of objects placed at non-decision points in our group of healthy older adults. Furthermore, this effect was in part modulated by attention in the controls: for objects placed at decision points, no effect of attention was found, whereas for objects placed at non-decision points the highly attended objects (i.e., the toys) were remembered better than the less attended objects (i.e., the non-toys). Interestingly, the AD patients showed a somewhat different result. First, for the non-decision point objects, attended items were more accurately recognized than less attended items, although recognition accuracy for these two stimulus types did not differ significantly from chance performance. However, for the objects placed at decision points, the unattended objects were remembered more accurately and well above chance level compared to the attended objects (for which only a marginally significant above-chance performance was found). Moreover, AD patients were more accurate for the unattended objects at decision points when directly compared to attended objects placed at decision points.

These results extend previous findings on landmark recognition in AD patients. For example, Cherrier et al. [31] made a distinction between memory for landmarks and non-landmarks that were presented along an actual route the patients had to walk. They showed that AD patients performed worse than controls with respect to landmark recognition, but performed better on landmark recognition compared to non-landmark recognition. However, it can be argued that this is the results of landmarks attracting more attention than non-landmarks. In contrast, it has also been suggested that Alzheimer patients have a heightened threshold for detecting stimuli in the environment [32]. While it could be argued that this spared memory for landmarks is due to more attention being directed at objects at decision points, our results clearly counter this explanation, since highly attended landmarks were remembered worse than less attended landmarks in AD.

An explanation for this may be that attentional resources allocated to objects cannot be recruited for the contextual processing (i.e., integrating the object information to spatial information). In unattended objects, cognitive resources can be fully allocated to object-space integration, required for transforming an object relevant for navigation into a true landmark. Indeed, there is evidence that object-location binding requires cognitive resources and does not occur automatically [33], unlike the processing of the spatial information itself, which may be an automatic, cognitively less demanding process [34]. This suggestion may explain why the unattended landmarks are remembered better than the attended landmarks in AD patients. Conversely, in the healthy older adults, attentional resources can be effectively allocated to both object processing and object-space binding, hence showing no performance difference between the attended and unattended landmarks.

With respect to the underlying neurocognitive mechanisms, previous fMRI studies have consistently demonstrated parahippocampal activation related to memory for landmarks in this task. Although our patient group is characterized by atrophy in the medial temporal lobe characteristic for AD even in the early stages of the disease, it is difficult to disentangle the involvement of specific brain areas in the present study. However, it is likely that this atrophy extends from the hippocampus proper to the adjacent parahippocampal gyrus. Although both the parahippocampal gyrus and the hippocampus have been found to be implicated in mnemonic object-location binding [35], [36], a previous study investigating object-location memory in virtual rooms has demonstrated spared implicit memory for object-location as well in AD patients [37]. Possibly, non-MTL brain regions, such as the basal ganglia [38] or the peristriate cortex [39], may compensate for functional loss in the hippocampal area in AD patients. While it was not our aim to examine memory for the navigational aspects themselves, several recent studies that examined route learning in AD patients demonstrated that already in the early stages of the disease spatial learning is impaired, both with respect to forming allocentric and egocentric representations [40]–[43]. Our present results, however, clearly show that navigationally-relevant object information can still be processed and stored into memory in AD patients with MTL atrophy. They also provide important information for cognitive rehabilitation in AD patients as attentional processes are not necessary and might even hamper successful information coding.
